# CancerEST: a web-based tool for automatic meta-analysis of public EST data

**DOI:** 10.1093/database/bau024

**Published:** 2014-04-07

**Authors:** Julia Feichtinger, Ramsay J. McFarlane, Lee D. Larcombe

**Affiliations:** ^1^North West Cancer Research Institute, Bangor University, Bangor, Gwynedd LL57 2UW, UK, ^2^Institute for Genomics and Bioinformatics, Graz University of Technology, Petersgasse 14, 8010 Graz, Austria, ^3^Core Facility Bioinformatics, Austrian Centre of Industrial Biotechnology, Petersgasse 14, 8010 Graz, Austria, ^4^NISCHR Cancer Genetics Biomedical Research Unit, Bangor University, Bangor, Gwynedd LL57 2UW, UK, ^5^Liverpool Cancer Research UK Centre, University of Liverpool, Liverpool, Merseyside L3 9TA, UK and ^6^Applied Mathematics and Computing Group, Cranfield University, Cranfield, Bedfordshire MK43 0AL, UK

## Abstract

The identification of cancer-restricted biomarkers is fundamental to the development of novel cancer therapies and diagnostic tools. The construction of comprehensive profiles to define tissue- and cancer-specific gene expression has been central to this. To this end, the exploitation of the current wealth of ‘omic’-scale databases can be facilitated by automated approaches, allowing researchers to directly address specific biological questions. Here we present CancerEST, a user-friendly and intuitive web-based tool for the automated identification of candidate cancer markers/targets, for examining tissue specificity as well as for integrated expression profiling. CancerEST operates by means of constructing and meta-analyzing expressed sequence tag (EST) profiles of user-supplied gene sets across an EST database supporting 36 tissue types. Using a validation data set from the literature, we show the functionality and utility of CancerEST.

**Database URL**: http://www.cancerest.org.uk

## Introduction

Identifying novel candidate markers/targets is a key challenge in the development of cancer therapies (1). Tissue- and cancer-specific gene expression profiles provide information about the potential of genes to serve as clinical markers (2). Thus, accessible and automated approaches analyzing the current wealth of ‘omic’-scale data are required to facilitate the full exploitation of expression data. Expressed sequence tags (ESTs) are short DNA sequences (200–500 nucleotides) generated by sequencing the 5′ and/or 3′ ends of cDNAs that are subsequently clustered and counted (3). In the past decade, a large amount of EST data has been deposited in public repositories such as dbEST (4), which currently holds records of 8 692 773 human ESTs. Unigene has grouped these expression data into clusters and assigned them to genes, facilitating the indexing of the EST data (5). Pipelining the retrieval, the integration and the high-throughput investigation of such data in a fashion specifically tailored to the interests of the user should facilitate wider application by putting EST data in the hands of researchers directly addressing focused biological questions, without requiring the involvement of bioinformaticians. Integration and subsequent investigation of EST data can not only enhance reliability and generalizability of results but can also reveal a comprehensive expression profile across numerous tissues, which can be used to uncover information about tissue-specific expression, cancer expression and, above all, cancer marker/target potential (6). For example, Kim *et al.* (7) and Campagne and Skrabanek (8) identified potential cancer markers by means of EST data analyses, whereas Hofmann *et al.* (9) used EST data, reverse transcription polymerase chain reaction (RT-PCR) and other high-throughput gene expression data to evaluate the tissue specificity and the cancer gene expression profiles of previously published cancer testis (CT) genes, a group of genes widely used in clinical applications (10).

Here we present CancerEST, a freely accessible pipeline with a user-friendly and intuitive web interface to provide automated high-throughput investigation of public EST data with user-defined sets of biologically significant and related genes to determine (i) their cancer marker/target potential, (ii) their tissue specificity and (iii) their comprehensive expression profiles across 36 tissues (Supplementary Table S1). The underlying method was developed for a previously published study, where we identified a cohort of novel cancer-specific marker genes (11), and has been improved and automated to provide the basis of CancerEST. The tool provides intuitive data analysis and visualizations and allows biologists/clinicians without skills in bioinformatics to exploit the wealth of publicly available data presented by modern databases. It serves to focus the overwhelming number of putative target genes on a manageable number of candidates, which can be followed up in the laboratory. To validate our approach, we have analyzed a list of testis-restricted genes from literature (9) and could reproduce the published results.

## Methods and structure of CancerEST

**Figure 1.**
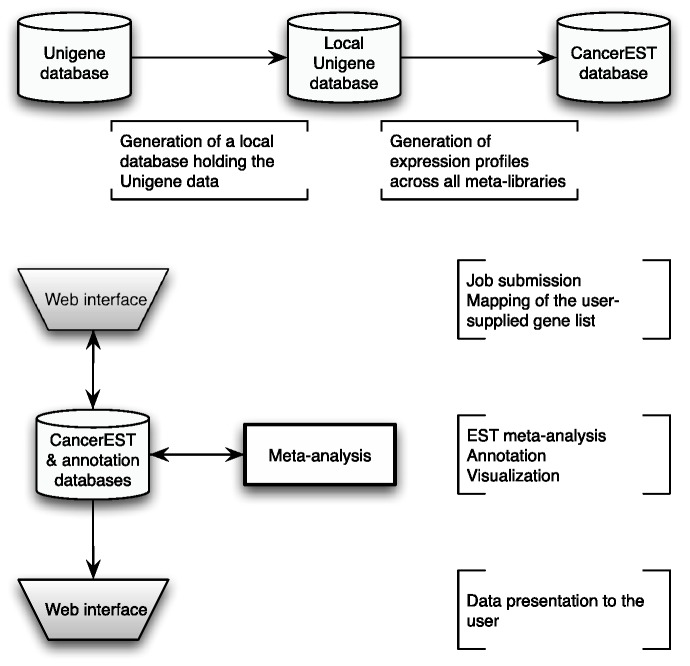
CancerEST workflow. The complete Unigene database was established as a local MySQL database and subsequently used to construct meta-libraries for 36 tissue types, allowing the computation of integrated expression profiles for all genes with assigned Unigene clusters. The web interface box indicates the areas where the user provides input and/or can view the mapping or analysis results. The analysis is carried out automatically without any user input and computes integrated expression profiles tailored to the interests of the user with visualizations to aid the data interpretation.

CancerEST consists of a web interface, pipelined analyses and three relational databases; one holding the analysis data, one holding the Unigene data and another one holding the gene annotation data. The principal workflow is shown in Figure 1.

### The CancerEST web interface

First, the CancerEST web interface handles the user specifications and mapping of the user-supplied gene list as well as the job submission. Second, it allows the user to view and download the analysis results and visualizations.

When submitting a new job, the user provides a text file consisting of Unigene Cluster IDs, Entrez IDs or curated gene names, for which the identifiers are then mapped to their appropriate Unigene Cluster IDs to show the user which genes can be fed into the analysis. Furthermore, the user has to specify a tissue focus, where submitted genes are allowed to show expression in normal individuals; for example, the testis might be of interest to the user, as it is an immunologically privileged tissue (12). The user can optionally select an interfering tissue(s), where submitted genes are tolerated to show additional expression in normal individuals; for example, brain tissue could be selected by the user, as various genes that have been originally assumed to be testis-restricted are also expressed in the brain, another tissue residing in immunological privilege (13). Finally, the job can be submitted by providing an email address.

When viewing a finished job, the results of the analysis and the visualizations are presented to the user in a simple-to-use web interface. All result files are also available for download. The web site makes use of cookies to ensure that a user only has access to his/her own data and thus can access secure areas of the web site (refer to the CancerEST help section available at http://www.cancerest.org.uk/help.html for more information). We also provide an example data set on our web site (available at http://www.cancerest.org.uk).

### EST data Retrieval, data quality and CancerEST databases

We obtained the complete data available from the Unigene database (Unigene Build #230) (5) and set up a local MySQL database. We excluded ESTs from normalized and subtracted cDNA libraries (6) as well as cDNA libraries deriving from uncharacterized, mixed or embryonic/fetal tissues. The exclusion of cancer cell-line libraries is optional and can be specified by the user. Furthermore, we kept only libraries from cancerous and healthy tissues, and thus excluded libraries deriving from diseases other than cancer. All ESTs of a given tissue type *t* were merged to a meta-library. However, meta-libraries with a combined EST count for healthy and cancerous tissues below 10 000 were excluded to ensure significance, resulting in cancer and normal meta-libraries for 36 tissue types (Supplementary Table S1, Supplementary Figure S1). For each Unigene cluster, the global expression profile in cancerous and healthy tissues is computed by EST counting, following the concept of the Unigene EST profiles (5). The expression profiles in cancerous and healthy tissues are normalized by calculating the transcripts per million *tpm_t,c_*, where *m_t,c_* is the number of ESTs for a given cluster *c* and for a given tissue type *t*, and *n_t_* is the total number of ESTs for that given tissue type *t*:

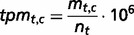



For annotation purposes, the Ensembl database (14) and the HUGO Gene Nomenclature Committee(HGNC) database (15) were established as a local MySQL database.

### The CancerEST pipeline

The pipeline handles the EST meta-analysis, the annotation and the visualizations. For each of the submitted genes, the expression profile is examined to determine the expression in the user-specified tissue focus, in possible interfering tissues, in all other healthy tissues as well as in all cancer-derived tissues. Thus, the weighted average *tpm_av_* for these four tissue groups is computed, where *w_t_* is the weight of the given tissue *t* belonging to the set of tissues *g*, represented by the size of the meta-library:

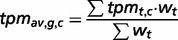



Genes are sorted into four classes according to their expression profile to provide information about their potential as cancer antigen-encoding genes: (i) tissue focus-restricted expression in normal individuals as well as cancer expression (Class 1), (ii) tissue focus- and interfering tissue-restricted expression in normal individuals as well as cancer expression (Class 2), (iii) tissue focus- and/or interfering tissue-restricted expression in normal individuals but no cancer expression (Class 3) and (iv) somatic expression in normal individuals (Class 4). The classes are designated with an ‘a’ if no focus expression was found.

The genes are also sorted into four states to provide information about tissue specificity: (i) tissue-specific (Classes 1–3), (ii) highly tissue-selective (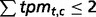 for all other healthy tissues), (iii) tissue-selective (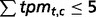 for all other healthy tissues) and (iv) enriched (the *tpm_av__,c_* of the tissue focus is twice the *tpm_t__,c_* of each of the other healthy tissues).

To evaluate the upregulation of genes of interest in cancer, the significance of upregulation is accessed using Fisher’s exact test (16). Genes with a p-value <0.05 or with expression in cancerous meta-libraries but not in the corresponding healthy meta-libraries are considered to be upregulated in these cancer types.

To visualize the analysis results, Circos plots (17) and bar charts are created. All data belonging to a user are stored for 30 days in the CancerEST user database, which can be accessed using the web interface during this time. This analytical approach was developed for a previous study published by the authors (11) and improved and automated for the basis of the CancerEST tool.

## Implementation

CancerEST runs on an Intel core i7 2.66-Ghz workstation with 12 GB RAM and is installed with CentOS 5.4 GNU Linux OS (x86_64). MySQL 5.0.77 (available at http://www.mysql.com) was used for the relational databases. The CancerEST web interface was implemented using HyperText Markup Language (HTML)/Cascading Style Sheets (CSS), Twitter Bootstrap (available at http://twitter.github.com/bootstrap/), Javascript/jQuery (available at http://jquery.com/) and Perl 5.8.8 (available at http://www.perl.org). The CancerEST pipeline was implemented using Perl 5.8.8 (available at http://www.perl.org). CancerEST is freely available online at http://www.cancerest.org.uk.

### Use of CancerEST

CancerEST was developed as a user-friendly and intuitive tool to compute cancer marker/target potential as well as to obtain comprehensive expression profiles and information about the tissue specificity for genes of interest to biologists/clinicians. The CancerEST web interface for viewing the analysis results consists of three sections: the overview, the information and the result section. The overview section provides basic information about the submitted job and a brief explanation on how to interpret the results. The information section includes, among others, the annotated genes of interest and the 36 tissue types supported by CancerEST. The result section includes the EST meta-analysis results comprising a ranked list of genes according to (i) their cancer marker potential, or (ii) their tissue specificity. Furthermore, a comprehensive expression profile across 36 healthy and cancerous tissues is available for each gene. Circos plots visualize the analysis results in their entirety to highlight relationships between the genes and the cancer types. In contrast, bar charts show the complete expression profile across 36 healthy and cancerous tissues for each gene separately. For more information, the CancerEST help section provides detailed documentation, available at http://www.cancerest.org.uk/help.html.

### Validation

**Figure 2.**
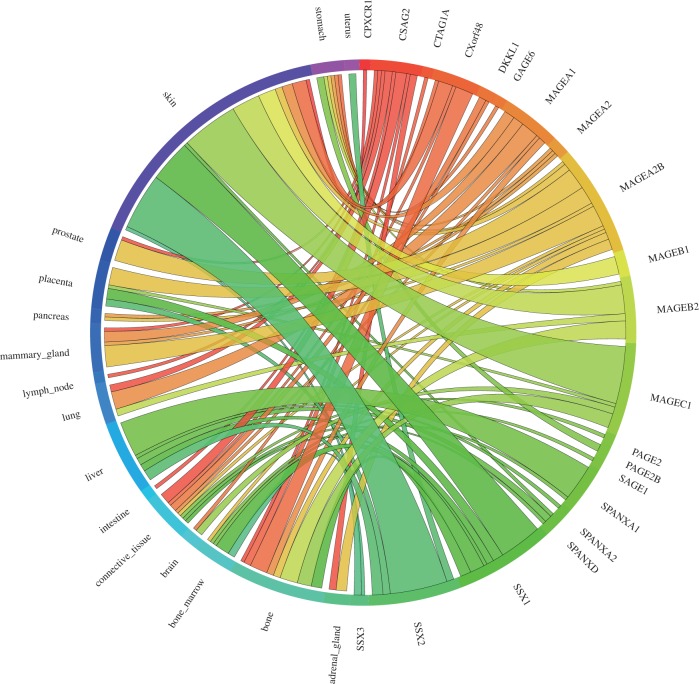
Circos plot showing the gene expression in relation to the corresponding cancer types for the 39 testis-restricted genes determined by Hofmann *et al.* (**9**). 21 of the 39 testis-restricted genes exhibit expression in various cancer types, in particular in melanoma (labelled as skin). Each connection between a gene and a cancer type indicates expression in a cancer. The magnitude of the connection corresponds to the transcripts per million (*tpm*) for the given gene in a given tissue.

**Figure 3.**
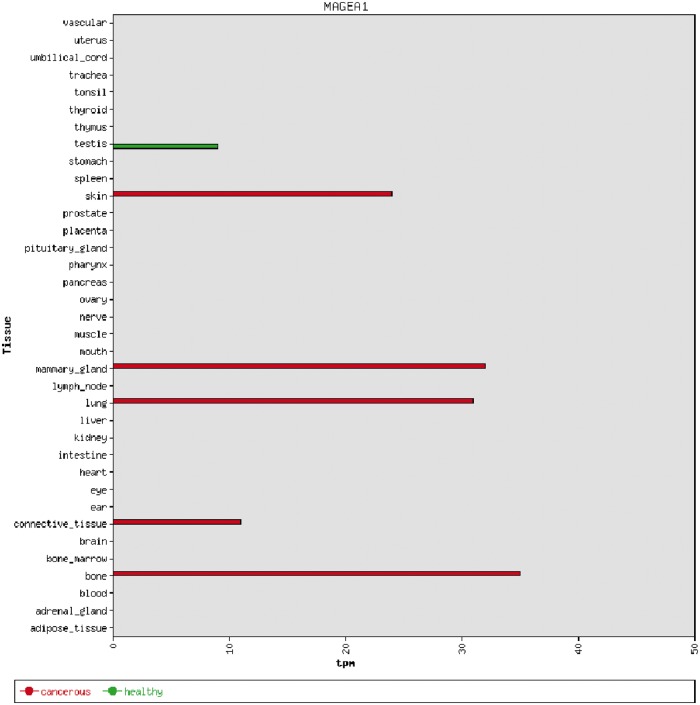
An example of a bar chart showing the integrated expression profile of the MAGEA1 gene. MAGEA1 exhibits a testis-restricted gene expression profile, but is aberrantly expressed in a number of cancer types. The expression is given in transcripts per million (*tpm*).

We used the 39 tight testis-restricted genes determined by Hofmann *et al.* as a validation data set (four genes could not be mapped to a Unigene cluster ID or to an HGNC gene name, resulting in 35 genes that could be evaluated). Hofmann *et al.* have evaluated the tissue- and cancer-specific expression of 153 CT genes previously published in the CTdatabase (18) using high-throughput expression data in combination with RT-PCR data (9). We selected ‘testis’ as tissue focus and chose ‘brain’ as interfering tissue, as it has been shown that various CT genes also exhibit expression in brain tissue (13). To be in accordance with Hofmann *et al.*, we additionally allowed placental gene expression and included cancer cell-line libraries. CancerEST determined 25 of these genes as not expressed in any healthy tissue or as tight testis-restricted (Supplementary Table S2). Additionally, seven genes were found to show limited evidence for brain expression, which could have been below the threshold of Hofmann *et al.*; however, these seven genes are not expressed in any other healthy tissue, consistent with Hofmann *et al.* The remaining three genes exhibit expression in other healthy tissues, although two of the genes show expression in only one, and one of the genes in only two other healthy tissues. Hofmann *et al.* detected cancer expression for 21 of the 35 testis-restricted genes. CancerEST reported cancer expression for 20 of these 21 genes and additionally predicted cancer expression for *GAGE6* (Supplementary Table S2), which has also been reported in the literature (19). In total, CancerEST predicted that 19 genes have high cancer marker/target potential by exhibiting a testis- or testis–brain-restricted expression profile as well as cancer expression (Figure 2, Supplementary Table S2). For example, the gene *MAGEA1*, which encodes the first CT genes to be discovered (20), is, according to CancerEST, expressed in various cancers including melanoma, lung cancer, breast cancer and bone and connective tissue sarcomas (Figure 3), an observation that is supported extensively through literature (21–25).

The results are consistent with Hofmann *et al.*; however, CancerEST uses a very stringent cutoff, which could explain the weak evidence for expression in the brain that was found for seven genes as well as the limited evidence for expression in healthy tissues that was found for three genes. Furthermore, with more EST data becoming available, the predictions become increasingly accurate, and CT genes originally believed to have testis-restricted expression profiles have to be adapted to testis-selective (9, 13). An alternative explanation for the limited evidence for expression in healthy tissues could be undiagnosed neoplastic change in the tissues analyzed, as many normal tissues are extracted from tissue obtained *post mortem* and are often pooled from tissues from a number of individuals, many of whom were aged at the time of death. In support of this, Chen *et al.* found discrepancies concerning the expression of some genes in normal tissues, as they detected expression in tissues from one panel of normal tissues, but could not detect expression in similar tissue types from a distinct second source (26). Thus, genes with testis-selective profiles could indeed be suitable candidates and be of clinical use.

In conclusion, tissue specificity was predicted accurately in 71% of the cases, including the genes showing expression in the brain even in 91% of the cases. Cancer expression was predicted correctly in 95% of the cases. Furthermore, Hofmann *et al.* reported that the widest range of CT gene expression was found in melanoma (9), which is consistent with our results (Figure 2) and the literature (27).

In our previous work (11) we have analyzed human meiotic genes using the approach now implemented into CancerEST and, with RT-PCR experimental validation and microarray meta-analysis, identified a novel, clinically relevant subgroup of the CT gene family (the meiCT genes), whose associated proteins have potential as novel cancer markers and therapeutic targets. This work can serve as an example workflow for potential users as well as a further validation data set. The developed microarray meta-analysis was also automated and implemented as a web tool (28) and is a complementary tool to CancerEST in identifying candidate cancer markers/targets, allowing the investigation of cancer expression in clinically relevant data. Furthermore, based on CancerEST and CancerMA analyses, a paper has been published, providing evidence that tumorigenesis in metazoans may involve a soma-to-germline transition, which could contribute to the acquisition of neoplastic characteristics (29). This work demonstrates the use of this tool and may guide the user toward new analysis possibilities using both CancerEST and CancerMA.

## Discussion

### Purposes and benefits of CancerEST

As tissue-specific gene expression plays a fundamental role in human biology and disease, the identification of genes with restricted/specific expression patterns helps to understand development, function and homeostasis of the distinct cell/tissue types as well as etiology, gene–tissue relationships and gene functions, thus aiding the discovery of novel marker/target genes (30–32). However, establishing a comprehensive map of tissue-specific expression for the complete human body poses an immense challenge owing to the difficulty of obtaining such data empirically, but can be facilitated by combining publicly available high-throughput expression data. CancerEST allows the automated construction of integrated expression profiles based on EST data across 36 tissues and thus can examine the tissue specificity as well as identify suitable cancer marker/therapeutic targets for a set of genes of interest, as shown by our validation. CancerEST permits users to focus on a manageable number of candidate genes, which can be followed up in the laboratory and thus decreases the risk to pursue unsuitable targets. The putative candidate genes could be used for diagnostic, therapeutic and prognostic strategies for specific cancer types, or to uncover common dysfunction of gene modules across various cancer types. Here, it should be noted that CancerEST is a tool to identify putative markers based on their expression profile and thus these candidate genes need to be further investigated by protein expression analyses of their associated proteins. Analyzing a set of co-expressed, co-regulated, interacting or otherwise related genes, however, can point to conserved disrupted pathways or mechanisms in cancer, as mutations in a vast number of genes have been associated with cancer, yet disruption of only a few key pathways may give rise to the characteristics of cancer (33).

### Comparison to databases and tools currently available

Several tools exist that exploit EST data to construct integrated expression profiles; for example, TissueInfo (34) and TiGER (35) determine the tissue specificity for a given gene or tissue-specific genes for a given tissue, but do not evaluate cancer expression or cancer marker/target potential, and, importantly, neither allow the analysis for sets of genes. In contrast, the Unigene tool Digital Differential Display (DDD) (5) compares EST profiles of user-defined EST libraries to identify genes with significantly different expression levels and another Unigene tool the EST Profile Viewer (5) shows the approximate expression profile for a given gene. However, neither of the two focuses on the cancer marker/target potential for a set of related genes. Several other tools were published but appear to be currently unavailable [DigiNorthern (36), ZooDDD (37), GBA server (38)]. Therefore, a simple-to-use web tool such as CancerEST computing the cancer marker/target potential, the tissue specificity as well as comprehensive expression profiles for a set of genes of interest to biologists/clinicians is not available to our knowledge.

## Conclusion

In summary, we present CancerEST, an integrated bioinformatic analytical pipeline that was used to automate the identification of novel candidate cancer markers/targets and/or to determine the tissue specificity by means of constructing and analyzing the EST expression profiles of user-supplied gene lists across 36 tissue types. Furthermore, such an automated pipeline with a simple-to-use web interface puts an integrated EST analysis in the hands of researchers who are directly addressing biological questions.

## Supplementary Data

Supplementary data are available at *Database* Online.

## References

[1] Ludwig JA, Weinstein JN (2005). Biomarkers in cancer staging, prognosis and treatment selection. Nat. Rev. Cancer.

[2] Backus J, Laughlin T, Wang Y (2005). Identification and characterization of optimal gene expression markers for detection of breast cancer metastasis. J. Mol. Diagn..

[3] Adams MD, Kelley JM, Gocayne JD (1991). Complementary DNA sequencing: expressed sequence tags and human genome project. Science.

[4] Boguski MS, Lowe TM, Tolstoshev CM (1993). dbEST—database for “expressed sequence tags”. Nat. Genet..

[5] Pontius JU, Wagner L, Schuler GD (2003). UniGene: a unified view of the transcriptome. The NCBI Handbook.

[6] Fierro AC, Vandenbussche F, Engelen K (2008). Meta analysis of gene expression data within and across species. Curr. Genomics.

[7] Kim B, Lee HJ, Choi HY (2007). Clinical validity of the lung cancer biomarkers identified by bioinformatics analysis of public expression data. Cancer Res..

[8] Campagne F, Skrabanek L (2006). Mining expressed sequence tags identifies cancer markers of clinical interest. BMC Bioinformatics.

[9] Hofmann O, Caballero OL, Stevenson BJ (2008). Genome-wide analysis of cancer/testis gene expression. Proc. Natl. Acad. Sci. USA.

[10] Simpson AJG, Caballero OL, Jungbluth A (2005). Cancer/testis antigens, gametogenesis and cancer. Nat. Rev. Cancer.

[11] Feichtinger J, Aldeailej I, Anderson R (2012). Meta-analysis of clinical data using human meiotic genes identifies a novel cohort of highly restricted cancer-specific marker genes. Oncotarget.

[12] Fijak M, Meinhardt A (2006). The testis in immune privilege. Immunol. Rev..

[13] Scanlan MJ, Gordon CM, Williamson B (2002). Identification of cancer/testis genes by database mining and mRNA expression analysis. Int. J. Cancer.

[14] Flicek P, Amode MR, Barrell D (2011). Ensembl 2012. Nucleic Acids Res..

[15] Seal RL, Gordon SM, Lush MJ (2011). genenames.org: the HGNC resources in 2011. Nucleic Acids Res..

[16] Fisher RA (1954). Statistical Methods for Research Workers. Biological Monographs and Manuals.

[17] Krzywinski M, Schein J, Birol I (2009). Circos: an information aesthetic for comparative genomics. Genome Res..

[18] Almeida LG, Sakabe NJ, deOliveira AR (2009). CTdatabase: a knowledge-base of high-throughput and curated data on cancer-testis antigens. Nucleic Acids Res..

[19] De Backer O, Arden KC, Boretti M (1999). Characterization of the GAGE genes that are expressed in various human cancers and in normal testis. Cancer Res..

[20] van der Bruggen P, Traversari C, Chomez P (1991). A gene encoding an antigen recognized by cytolytic T lymphocytes on a human melanoma. Science.

[21] Brasseur F, Rimoldi D, Liénard D (1995). Expression of MAGE genes in primary and metastatic cutaneous melanoma. Int. J. Cancer.

[22] Jang SJ, Soria JC, Wang L (2001). Activation of melanoma antigen tumor antigens occurs early in lung carcinogenesis. Cancer Res..

[23] Otte M, Zafrakas M, Riethdorf L (2001). MAGE-a gene expression pattern in primary breast cancer. Cancer Res..

[24] Sudo T, Kuramoto T, Komiya S (1997). Expression of MAGE genes in osteosarcoma. J. Orthop. Res..

[25] Antonescu CR, Busam KJ, Iversen K (2002). MAGE antigen expression in monophasic and biphasic synovial sarcoma. Hum. Pathol..

[26] Chen YT, Scanlan MJ, Venditti CA (2005). Identification of cancer/testis-antigen genes by massively parallel signature sequencing. Proc. Natl. Acad. Sci. USA.

[27] Scanlan MJ, Simpson AJG, Old LJ (2004). The cancer/testis genes: review, standardization, and commentary. Cancer Immun..

[28] Feichtinger J, McFarlane RJ, Larcombe LD (2012). CancerMA: a web-based tool for automatic meta-analysis of public cancer microarray data. Database.

[29] Feichtinger J, Larcombe L, McFarlane RJ (2014). Meta-analysis of expression of l(3)mbt tumor-associated germline genes supports the model that a soma-to-germline transition is a hallmark of human cancers. Int. J. Cancer.

[30] Su AI, Wiltshire T, Batalov S (2004). A gene atlas of the mouse and human protein-encoding transcriptomes. Proc. Natl. Acad. Sci. USA.

[31] Chikina MD, Huttenhower C, Murphy CT (2009). Global prediction of tissue-specific gene expression and context-dependent gene networks in *Caenorhabditis elegans*. PLoS Comput. Biol..

[32] Ramaswamy S, Tamayo P, Rifkin R (2001). Multiclass cancer diagnosis using tumor gene expression signatures. Proc. Natl. Acad. Sci. USA.

[33] Vogelstein B, Kinzler KW (2004). Cancer genes and the pathways they control. Nat. Med..

[34] Skrabanek L, Campagne F (2001). TissueInfo: high-throughput identification of tissue expression profiles and specificity. Nucleic Acids Res..

[35] Liu X, Yu X, Zack DJ (2008). TiGER: a database for tissue-specific gene expression and regulation. BMC Bioinformatics.

[36] Wang J, Liang P (2003). DigiNorthern, digital expression analysis of query genes based on ESTs. Bioinformatics.

[37] Chen YC, Hsiao CD, Lin WD (2006). ZooDDD: a cross-species database for digital differential display analysis. Bioinformatics.

[38] Wu X, Walker MG, Luo J (2005). GBA server: EST-based digital gene expression profiling. Nucleic Acids Res..

